# Renal function in patients with non-dialysis chronic kidney disease receiving intravenous ferric carboxymaltose: an analysis of the randomized FIND-CKD trial

**DOI:** 10.1186/s12882-017-0444-6

**Published:** 2017-01-17

**Authors:** Iain C. Macdougall, Andreas H. Bock, Fernando Carrera, Kai-Uwe Eckardt, Carlo Gaillard, David Van Wyck, Yvonne Meier, Sylvain Larroque, Simon D. Roger, on behalf of the FIND-CKD Study investigators, Simon D. Roger, Alastair Gilles, Randall Faull, Nigel D. Toussaint, Lawrence McMahon, Michael Suranyi, David Mudge, Brian Hutchison, Ashley Irish, Peter Kerr, Hemant Kulkarni, Grahame Elder, Margaret Jardine, Karl Lhotta, Gert Mayer, Raymond Vanholder, Bart Dirk Maes, Pieter Evenepoel, Frédéric Debelle, Michel Jadoul, Max Dratwa, Igor Macel, Milan Dunaj, Milan Kvapil, Petr Bucek, Jitka Rehorova, Ales Hruby, Václava Honová, Lada Malanova, Martin Lucak, Dalibor Lecian, Martin Jirovec, Jiri Vlasak, Ivan Rychlik, Stanislav Surel, Anne-Lise Kamper, Ove Ostergaard, Gudrun K. Steffensen, Leila Chenine, Gabrial Choukroun, Philippe Zaoui, Christoph Wanner, Wolfgang Backs, Uwe Kraatz, Frank Dellanna, Klaus Busch, Tobias Marsen, Wolfgang Seeger, Rainer Woitas, Nicholas Obermueller, Thomas Haak, Stephan Lueders, Frank Pistrosch, Eckhard Mueller, Peter R. Mertens, Werner Sutermer, Scott-Oliver Grebe, Syrus Hafezi-Rachti, Silke Roeser, Dimitrios Tsakiris, Dimitrios Memmos, Demetrios Vlachakos, Vassilis Vargemezis, Ioannis Stefanidis, Christos Syrganis, Polichronis Alivanis, Ioannis Papadakis, Nickolaos Papagalanis, Aimilios Andrikos, Dimitrios Goumenos, Kostas Siamopoulos, Charikelia Gouva, Gabriel Papadakis, Ioannis Boletis, Myrsini Tsimnadi-Spanoudaki, Dimitrios Stamatiades, Kyriaki Stamatelou, Spyridon Moutafis, Francesco Locatelli, Antonio Santoro, Francesco Quarello, Giuseppe Remuzzi, Salvatore Coppola, Rosella Ferraro Mortellaro, Andrea Icardi, Giacomo Colussi, Franco Della Grotta, Luigi Lombardi, Maurizio Gallieni, Giuseppe Villa, Giuseppe Grandaliano, Carlo Gaillard, Sebastiaan Huisman, Jos Barendregt, Peter J. H. Smak Gregoor, Cecilia Oien, Boleslaw Rutkowski, Robert Malecki, Michal Nowicki, Przemyslaw Rutkowski, Kryzsztof Marczewski, Michal Mysliwiec, Antoni Sydor, Jacek Rysz, Andrzej Rydzewski, Marian Klinger, Rafal Wnuk, Piotr Kozminski, Anna Nocon, Kazimierz Ciechanowski, Pedro Correia, Fernando Neves, José Barata, Gabriel Mircescu, Mihai Voiculescu, Gheorghe Gluhovschi, Eugen Mota, Angel Luís Martín De Francisco, Alberto Torre, Alba Herreros, José Luño, E Gruss, Judith Martins, Marti Vallés, Julio Pascual, Peter Bárány, Andreas H. Bock, Patrice M. Ambuehl, Sehsuvar Erturk, Mustafa Arici, Saime Paydas, Zeki Soypacaci, Taner Camsari, Sedat Ustundag, Iain C. Macdougall, Mark E. Thomas, Richard J. D’Souza, Jo E. Taylor, Nicholas R. Pritchard, Robin Jeffery, Stephen G. Riley, Deepak Bhatnagar, Sunil Bhandari, David Reaich, Paul E. Stevens, Mohsen El Kossi, Simon Roe, Brian Camilleri, Aimun Ahmed, Arif Khwaja, Barbara Thompson, Debasish Banerjee, Johann Nicholas, Alistair Hutchison, Richard Borrows

**Affiliations:** 1 Department of Renal Medicine, King’s College Hospital, Denmark Hill, London, SE5 9RS UK; 2 Department of Nephrology, Kantonsspital Aarau, Aarau, Switzerland; 3 Eurodial, DaVita, Leiria, Portugal; 4 Department of Nephrology and Hypertension, University of Erlangen-Nürnberg, Erlangen, Germany; 5 Department of Nephrology, University Medical Center Groningen, University of Groningen, Groningen, The Netherlands; 6 DaVita Healthcare Partners Inc., Denver, CO USA; 7 Vifor Pharma, Glattbrugg, Switzerland; 8 Renal Research, Gosford, NSW Australia

**Keywords:** Chronic kidney disease, Ferinject, Ferric carboxymaltose, eGFR, Intravenous, Renal function

## Abstract

**Background:**

Preclinical studies demonstrate renal proximal tubular injury after administration of some intravenous iron preparations but clinical data on renal effects of intravenous iron are sparse.

**Methods:**

FIND-CKD was a 56-week, randomized, open-label, multicenter study in which patients with non-dialysis dependent chronic kidney disease (ND-CKD), anemia and iron deficiency without erythropoiesis-stimulating agent therapy received intravenous ferric carboxymaltose (FCM), targeting either higher (400–600 μg/L) or lower (100–200 μg/L) ferritin values, or oral iron.

**Results:**

Mean (SD) eGFR at baseline was 34.9 (11.3), 32.8 (10.8) and 34.2 (12.3) mL/min/1.73 m^2^ in the high ferritin FCM (*n* = 97), low ferritin FCM (*n* = 89) and oral iron (*n* = 167) groups, respectively. Corresponding values at month 12 were 35.6 (13.8), 32.1 (12.7) and 33.4 (14.5) mL/min/1.73 m^2^. The pre-specified endpoint of mean (SE) change in eGFR from baseline to month 12 was +0.7 (0.9) mL/min/1.73 m^2^ with high ferritin FCM (*p* = 0.15 versus oral iron), -0.9 (0.9) mL/min/1.73 m^2^ with low ferritin FCM (*p* = 0.99 versus oral iron) and -0.9 (0.7) mL/min/1.73 m^2^ with oral iron. No significant association was detected between quartiles of FCM dose, change in ferritin or change in TSAT versus change in eGFR. Dialysis initiation was similar between groups. Renal adverse events were rare, with no indication of between-group differences.

**Conclusion:**

Intravenous FCM at doses that maintained ferritin levels of 100–200 μg/L or 400–600 μg/L did not negatively impact renal function (eGFR) in patients with ND-CKD over 12 months versus oral iron, and eGFR remained stable. These findings show no evidence of renal toxicity following intravenous FCM over a 1-year period.

**Trial registrations:**

ClinicalTrials.gov NCT00994318 (first registration 12 October 2009).

**Electronic supplementary material:**

The online version of this article (doi:10.1186/s12882-017-0444-6) contains supplementary material, which is available to authorized users.

## Background

The use of iron therapy to manage renal anemia in patients with chronic kidney disease (CKD) has increased significantly in recent years [1], partly in response to concerns about the safety of erythropoiesis-stimulating agent (ESA) therapies [2, 3]. Randomized trials have shown intravenous (IV) iron therapy to be more effective than oral iron in terms of replenishing depleted iron stores and improving anemia in patients on dialysis [4–6]. In non-dialysis dependent CKD (ND-CKD), trials have confirmed the benefits of IV versus oral iron therapy but have typically been no longer than 8 weeks in duration [7–11]. Recently, the randomized 56-week FIND-CKD study compared IV ferric carboxymaltose (FCM) versus oral iron in patients with ND-CKD, anemia, and iron deficiency not receiving ESA therapy [12]. Intravenous FCM targeting a ferritin level of 400–600 μg/L delayed and/or reduced the need for other anemia management (including ESAs) significantly at 1 year compared to patients receiving oral iron, and the hematopoietic response was more rapid.

However, concerns exist about the potential renal toxicity of IV iron therapy [13]. Rapid release of large amounts of iron into the bloodstream could generate ‘free’ iron in the circulation (non-transferrin bound iron, NTBI) which may promote oxidative stress [14, 15]. Some IV iron complexes such as ferric gluconate contain weakly-bound iron that is released readily and quickly [15]. In contrast, animal models have shown, that oxidative stress does not increase with more stable IV iron complexes such as FCM [16–18]. Clinical evidence relating to a possible effect of IV iron therapy on renal function is limited. Single-dose and short-term (5-week) studies from one center have indicated that iron sucrose may induce renal injury mediated by oxidative stress and inflammation [19–23]. However, the recently published REVOKE study, which randomized patients with ND-CKD to IV iron sucrose or oral iron, showed neither a difference in renal function decline (based on GFR measured by iothalamate clearance) nor in proteinuria during follow-up lasting up to 2 years [24]. Confirmatory data are clearly important.

The FIND-CKD trial included protocol-specified monitoring of renal function in over 600 patients with ND-CKD, based on estimated GFR (eGFR), throughout the 1-year study [25]. Data were analyzed to compare renal outcomes in patients randomized to IV FCM using two different dosing regimens aiming for different target ferritin concentrations, with those in patients receiving oral iron.

## Methods

### Study design

FIND-CKD was a 56-week, open-label, multicenter, prospective, randomized, three-arm study undertaken during December 2009 to January 2012 at 193 nephrology centers in 20 countries (ClinicalTrials.gov NCT00994318) [25].

### Patient population

Adult patients (≥18 years) with ND-CKD were eligible for inclusion if (a) at least one Hb level was between 9 and 11 g/dL within 4 weeks of randomization, (b) any ferritin level was <100 μg/L, or <200 μg/L with transferrin saturation (TSAT) <20%, within 4 weeks of randomization, (c) eGFR was ≤60 mL/min/1.73 m^2^ (four-variable Modification of Diet in Renal Disease [MDRD-4] equation [26]), the prior rate of eGFR loss was ≤12 mL/min/1.73 m^2^/year and predicted eGFR at 12 months based on previous decline was ≥15 mL/min/1.73 m^2^, and (d) no ESA had been administered within 4 months prior to randomization. Estimates of prior eGFR loss were based on at least two values over at least 4 weeks prior to randomization, and preferably three values over at least 3 months.

Key exclusion criteria included current dialysis, anticipated dialysis or transplantation during the study, anemia due to reasons other than iron deficiency, a documented history of discontinuing oral iron products due to significant gastrointestinal distress, known active infection, C-reactive protein >20 mg/L, overt bleeding, active malignancy, chronic liver disease, concomitant New York Heart Association Class IV heart failure and poorly controlled hypertension (>160 mmHg systolic pressure or >100 mmHg diastolic pressure).

### Randomization and intervention

Eligible patients were randomized centrally via a central interactive voice-response system in a 1:1:2 ratio to high ferritin FCM, low ferritin FCM or oral iron. The dose of FCM (Ferinject^®^, Vifor International, St Gallen, Switzerland) in the high ferritin and low ferritin FCM groups was adjusted to target a ferritin level of 400−600 μg/L and 100–200 μg/L, respectively. An initial single dose was administered on day 0: 1000 mg iron as FCM in the high ferritin FCM group (500 mg iron on days 0 and 7 in patients weighing ≤66 kg) and 200 mg iron as FCM in the low ferritin FCM group if ferritin was <100 μg/L. During weeks 4 to 48, FCM was administered every 4 weeks in the high ferritin FCM group at a dose of 500 mg iron if ferritin was in the range 200 to <400 μg/L, and at a dose of 1000 mg iron if ferritin was <200 μg/L, and in the low ferritin FCM group at a dose of 200 mg iron if ferritin was <100 μg/L. In both groups, dosing was withheld if TSAT was ≥40%. Oral iron therapy consisted of commercially-available ferrous sulfate at a dose of 304 mg (100 mg of iron) twice daily to week 52. During the first 8 weeks after randomization, patients were not to receive ESAs, blood transfusion or any anemia therapy other than study drug unless there was an absolute requirement, after which ESAs and other therapies were permitted according to local practice if the Hb was <10 g/dL.

### Assessment of renal function

Renal function was assessed by eGFR, with values calculated locally and provided by the study sites using the MDRD-4 formula [26]. Estimated GFR was recorded at baseline and at every 3 months throughout the 12-month study period. The change in eGFR from baseline to the end of the study was a pre-specified secondary endpoint of the trial. GFR was estimated by the MDRD-4 formula [26]. As a *post hoc* sensitivity analysis, GFR was also estimated by the creatinine-based Chronic Kidney Disease Epidemiology Collaboration (CKD-EPI) formula [27]. CKD-EPI values were calculated centrally using locally-measured serum creatinine levels. The percentage of patients starting dialysis was a further pre-specified secondary endpoint.

### Statistical analysis

All analyses of renal function were exploratory. Analysis of covariance (ANCOVA) modeling was used to compare the change in eGFR values from baseline to month 12 between groups based on least square (LS) mean values using a repeated fixed effects model with treatment, visit and pooled country as factors, baseline eGFR as covariate, and treatment-by-visit as an interaction. Change in eGFR at month 12 was summarized in subpopulations of patients according to age (≤ or > median), gender, body mass index (BMI, ≤ or > median), baseline systolic and diastolic blood pressure (≤ or > median), mean arterial pressure and history of diabetes at baseline. Furthermore, a multivariate analysis including demographics and baseline characteristics (age, gender, BMI, systolic and diastolic blood pressure, diabetic status, prior use of angiotensin converting enzyme [ACE] inhibitor and angiotensin II receptor blocker [ARB] medications) was performed to check for potential confounding effect and best impacting factor on the analysis of treatment effect.

For the proportion of patients requiring dialysis, logistic regression analyses were performed and odds ratios (ORs) were used to compare treatment groups.

*Post hoc*, absolute eGFR values and the change in eGFR from baseline to month 12 were analyzed according to (i) quartiles of total FCM dose throughout the 12-month study using pooled data from both FCM treatment groups (ii) quartiles of change in ferritin level from baseline to month 12 across all patients (iii) quartiles of change in TSAT level from baseline to month 12 across all patients.

Renal function was analyzed in the intention-to-treat (ITT) population, comprising all patients who received at least one dose of randomized treatment and who attended at least one post-baseline visit. Patients were excluded from the analysis of change in eGFR to month 12 if (a) they reached the primary event before month 12 (i.e. received alternative management for anemia) or (b) the randomized treatment regimen was permanently discontinued before month 12. Within this cohort, calculations for the change in eGFR, ferritin and TSAT from baseline to month 12 were based on the subpopulations of patients who had values available at both time points.

Adverse events were analyzed in the safety population, comprising all patients who received at least one dose of randomized study drug.

All statistical analyses were performed using SAS Version 9.3 (SAS Institute Inc. SAS/STAT, Cary, NC, USA).

## Results

### Study population

In total, 613 patients were randomized and included in the ITT population. Estimated GFR was measured at baseline in all patients. Of 519 patients who completed the study, 166 patients had started another anemia management and/or stopped the randomized study regimen before month 12, and were excluded from analyses. Thus eGFR values at both baseline and month 12 were analyzed in 353 patients (97, 89 and 167 patients in the high ferritin FCM group, the low ferritin FCM group and the oral iron group, respectively). These patients were included in the current analysis. The demographics and other characteristics of this subpopulation (Table 1) did not differ from the total ITT population (Additional file 1: Table S1) and were comparable between groups.

**Table 1:** Baseline characteristics for patients with eGFR values at baseline and month 12

	High ferritin FCM (*n* = 97)	Low ferritin FCM (*n* = 89)	Oral iron (*n* = 167)
Age, years	69.3 (12.9)	69.0 (12.1)	69.6 (12.7)
Female gender, n (%)	61 ((62.9)	56 (62.9)	106 (63.5)
White race, n (%)	93 (95.9)	83 (93.3)	159 (95.2)
Body mass index, kg/m^2^	30.5 (6.8)	30.0 (5.3)	29.4 (5.4)
History of diabetes, n (%)	61 (62.9)	59 (66.3)	106 (63.5)
Endogenous erythropoietin, mIU/mL	29.4 (24.6)	29.6 (27.4)	26.3 (20.9)
Hb, g/dL	10.4 (0.7)	10.5 (0.9)	10.7 (0.6)
Ferritin, μg/L	54.2 94.9)	45.8 (44.3)	52.4 (39.9)
TSAT, %	16.3 (20.2)	14.9 (7.5)	14.8 (7.0)
C-reactive protein, mg/L	7.4 (13.4)	5.7 (5.9)	5.3 (6.5)
ACE inhibitor therapy prior to study entry, n (%)^a^	32 (33.0)	37 (41.6)	69 (41.3)
Angiotensin II antagonist therapy prior to study entry, n (%)^b^	41 (42.3)	33 (37.1)	77 (46.1)

Continuous variables are shown as mean (SD)

*ACE* angiotensin converting enzyme inhibitor, *FCM* ferric carboxymaltose, *Hb* hemoglobin, *TSAT* transferrin saturation

^a^Includes patients receiving ACE inhibitor combinations

^b^Includes patients receiving angiotensin II antagonist combinations

Baseline eGFR in this subpopulation of patients was similar between treatment groups (Table 2) and did not show any relevant differences to baseline values in the total ITT population (mean [SD] 32.8 [11.7] mL/min/1.73 m^2^, 31.5 [10.7] mL/min/1.73m^2^and 32.3 [11.6] mL/min/1.73 m^2^, respectively, in the high ferritin FCM, low ferritin FCM and oral iron groups).

**Table 2:** Estimated GFR (eGFR) for patients with eGFR values at baseline and month 12

	High ferritin FCM (*n* = 97)	Low ferritin FCM (*n* = 89)	Oral iron (*n* = 167)
eGFR at baseline (MDRD), mL/min/1.73 m^2^			
Mean (SD)	34.9 (11.3)	32.8 (10.8)	34.2 (12.3)
≥ 60, n (%)	1 (1.0)	1 (1.1)	3 (1.8)
30 to <60, n (%)	62 (63.9)	51 (57.3)	101 (60.5)
15 to <30, n (%)	34 (35.1)	37 (41.6)	60 (35.9)
< 15, n (%)	0	0	3 (1.8)
eGFR at month 12 (MDRD), mL/min/1.73 m^2^			
Mean (SD)	35.6 (13.8)	32.1 (12.7)	33.4 (14.5)
≥ 60, n (%)	7 (7.2)	4 (4.5)	7 (4.2)
30 to <60, n (%)	54 (55.7)	40 (44.9)	83 (49.7)
15 to <30, n (%)	32 (33.0)	39 (43.8)	65 (38.9)
< 15, n (%)	4 (4.1)	6 (6.7)	12 (7.2)
Change from baseline to month 12, LS mean (SE) (MDRD), mL/min/1.73 m^2^	0.7 (0.9)	-0.9 (0.9)	-0.9 (0.7)
*p* value for change vs oral iron^a^	0.15	0.99	Reference
Relative change from baseline to month 12, LS mean (SE) (MDRD), %	3.1 (2.6)	-2.4 (2.7)	-2.2 (2.0)
*p* value for change vs oral iron^a^	0.098	0.95	Reference
eGFR (CKD-EPI) mL/min/1.73 m^2^	*n* = 82	*n* = 68	*n* = 137
Mean (SD) at baseline	33.5 (11.9)	32.0 (11.8)	32.5 (13.4)
Mean (SD) at month 12	34.8 (13.1)	31.1 (13.5)	31.0 (14.8)
Change, LS mean (SE), mL/min/1.73 m^2^	1.3 (1.0)	-1.2 (1.0)	-1.7 (0.7)
*p* value for change vs oral iron^a^	0.012	0.68	Reference

eGFR was estimated by the MDRD-4 equation [27] at the local laboratory

*CI* confidence interval, *CKD-EPI* Chronic Kidney Disease Epidemiology Collaboration (CKD-EPI), *eGFR* estimated GFR, *FCM* ferric carboxymaltose, *LS* least squares, *MDRD* Modification of Diet in Renal Disease, *SE* standard error

^a^Analysis of covariance analysis based on least square mean values, using repeated measures

Four patients in this cohort of 353 patients were included against protocol with baseline eGFR >60 mL/min/1.73 m^2^: one high ferritin FCM patient (63 mL/min/1.73 m^2^), one low ferritin FCM patient (61 mL/min/1.73 m^2^) and two patients in the oral iron group (66 and 77 mL/min/1.73 m^2^). Three patients in the oral iron group contravened the exclusion criterion that eGFR loss was to be no more than 12 mL/min/1.73 m^2^ per year.

### Change in renal function

**Fig. 1:**
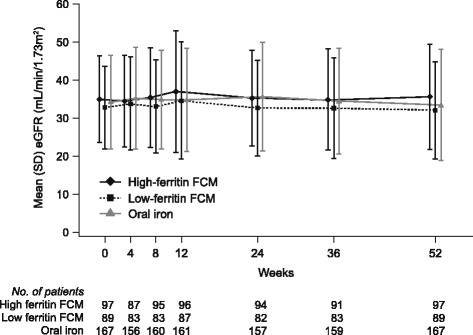
Estimated GFR to month 12 according to treatment group in patients with eGFR values at baseline and month 12. Values are shown as mean (SD). FCM, ferric carboxymaltose; eGFR, estimated GFR

Values for eGFR showed no change in any of the three treatment groups throughout the 12-month study (Fig. 1). At month 12, mean (SD) eGFR was 35.6 (13.8) mL/min/1.73 m^2^, 32.1 (12.7) mL/min/1.73 m^2^ and 33.4 (14.5) mL/min/1.73 m^2^, respectively. The pre-defined endpoint of change from baseline to month 12 was +0.7 (0.9) mL/min/1.73 m^2^ in the high ferritin FCM group, -0.9 (0.9) mL/min/1.73 m^2^ in the low ferritin FCM group and -0.9 (0.7) mL/min/1.73 m^2^ in the oral iron group (least square [LS] mean [SE] values). There were no significant differences in the change from baseline to month 12 for either FCM group versus oral iron (*p* = 0.15 for the high ferritin group, *p* = 0.99 for the low ferritin FCM group). The mean (SE) percentage change in eGFR was 3.1 (2.6)% in the high ferritin FCM group (*p* = 0.098 versus oral iron) (Table 2).

As a sensitivity analysis, eGFR was also calculated using the CKD-EPI formula. Serum creatinine values were provided for central calculation of CKD-EPI values in 82, 68 and 137 patients in the high ferritin FCM, low ferritin FCM and oral iron groups, respectively. Based on the CKD-EPI formula, there was a significant increase in eGFR from baseline to month 12 for the high ferritin FCM group versus oral iron (*p* = 0.012) (Table 2).

When the change in eGFR from baseline to month 12 was assessed in subpopulations of patients according to age, gender, BMI, presence/absence of diabetes, systolic and diastolic blood pressure and mean arterial pressure, no apparent influence of treatment group was observed (Additional file 2: Table 2).

### Change in renal function according to FCM dose

**Fig. 2:**
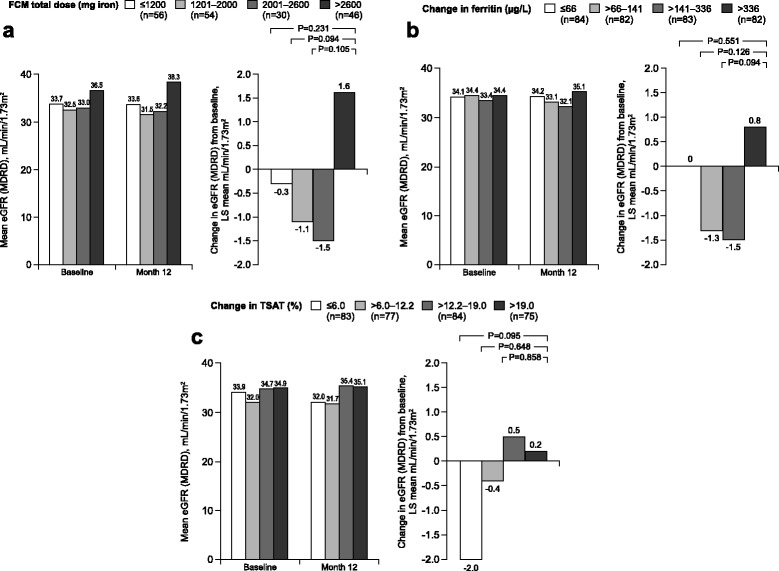
Absolute estimated glomerular filtration rate (eGFR) and change in eGFR from baseline to month 12 according to quartiles of (**a**) total ferric carboxymaltose (FCM) dose to month 12 in patients randomized to high ferritin FCM or low ferritin FCM (**b**) change in ferritin from baseline to month 12 across all treatment groups and (**c**) change in transferrin saturation (TSAT) from baseline at month 12 across all treatment groups. Data are shown for patients with eGFR values at baseline and month 12. Change in eGFR is shown as least squares (LS) mean values. MDRD, Modification of Diet in Renal Disease

The mean (SD) total dose of FCM 2793 (932) mg iron in the high ferritin group and 1205 (626) mg iron in the low ferritin group among patients with eGFR data available at baseline and month 12 (excluding patients who started another anemia therapy or permanently discontinued study treatment.) The change in eGFR from baseline to month 12 showed no association with total FCM dose when plotted individually for patients in either the high ferritin FCM or low ferritin FCM groups (Additional file 3: Figure S1). Using pooled data from both FCM groups, the change in eGFR to month 12 was analyzed by quartiles of total FCM dose (Fig. 2a).

The multivariate model indicated that age (*p* = 0.007), systolic blood pressure (*p* = 0.004), diabetic status (*p* = 0.058) and prior use of ACE inhibitor therapy (*p* = 0.054) exerted an impact on the change in eGFR (MDRD) to month 12. When these factors were added into the repeated measures model over time, the least square mean values for treatment effect were similar for the high ferritin FCM, low ferritin FCM and oral iron groups (0.7, -0.8 and -0.9 mL/min/1.73 m^2^, respectively; *p* = 0.14 for high ferritin FCM versus oral iron, *p* = 0.92 for low ferritin FCM versus oral iron). When repeated using the CKD-EPI equation to estimate GFR, only age (*p* = 0.042) and systolic blood pressure (*p* = 0.004) were found to influence the change in eGFR to month 12. Inclusion of these two factors in the repeated measure model produced LS mean values of 1.3 mL/min/1.73 m^2^ for the high ferritin FCM group, -1.1 mL/min/1.73 m^2^ for the low ferritin FCM group and -1.7 mL/min/1.73 m^2^ for the oral iron group (*p* = 0.010 versus high ferritin FCM, *p* = 0.613 versus low ferritin FCM).

### Change in renal function according to iron status

Mean ferritin levels were within the pre-specified target ranges from week 12 to the end of the study in both of the FCM treatment arms (Additional file 4: Figure S2a). At month 12, the mean (SD) change in ferritin from baseline was 455 (116), 81 (59) and 139 (111) μg/L in the high ferritin FCM, low ferritin FCM and oral iron groups, respectively, among patients with eGFR available at baseline and month 12. The change in eGFR from baseline to month 12 showed no significant association with the change in ferritin over the same period when analyzed by quartiles (Fig. 2b).

TSAT levels to month 12 are shown in Additional file 4: Figure S2b. As observed for ferritin levels, the change in eGFR showed no significant differences between quartiles of change in TSAT (Fig. 2c).

### Renal events

In total, 16/613 patients in the ITT population (2.6%) progressed to dialysis by month 12 (5 high ferritin FCM, 1 low ferritin FCM, 10 oral iron). There was no significant difference in the risk of dialysis for either FCM group versus oral iron: OR 1.01 (95% CI 0.34, 3.00; *p* = 0.99) for the high ferritin FCM group and OR 0.20 (95% CI 0.03, 1.56; *p* = 0.12) for the low ferritin group. No patient underwent renal transplantation.

Rates of adverse events and serious adverse events related to renal function were low with no indication of clinically relevant differences between treatment groups (Additional file 5: Table S3).

## Discussion

Results from the randomized FIND-CKD trial show that compared to oral iron, administration of IV FCM in doses that maintain ferritin levels of 100–200 μg/L or 400–600 μg/L does not negatively impact renal function, as determined by eGFR, in patients with ND-CKD after 1 year. Mean eGFR remained stable during the study in both the FCM treatment groups, and the change in eGFR to 1 year did not differ from that seen in patients treated with oral iron therapy, either on univariate or multivariate analysis. These findings, calculated using the pre-specified MDRD-4 formula, were confirmed when GFR was estimated by the more recently developed CKD-EPI formula [28]. Indeed, if anything, there was an increase in eGFR in the patients in the high-ferritin FCM arm compared to oral iron using the CKD-EPI formula (*p* = 0.012) which was confirmed on multivariate analysis. There was no difference between groups in the rate of progression to dialysis and no evidence of increased renal adverse events in either FCM treatment group.

Clinical studies measuring the short- or long-term effect of IV iron complexes on renal function versus controls are relatively scarce. Van Wyck et al. randomized 188 patients with ND-CKD to a total dose of 1000 mg iron as iron sucrose (infused over 3.5–4 h) or oral iron sulfate [10]. At the end of the 6-week study, there was a mean decrease in eGFR in both treatment arms, but the decrease was smaller in the iron sucrose arm (-1.45 mL/min/1.73 m^2^ versus -4.40 mL/min/1.73 m^2^ in the oral iron arm; *p* = 0.01). McMahon et al. undertook a randomized trial of iron sucrose (100–200 mg every two months) or oral iron sulfate for 12 months in patients with ND-CKD who were non-anemic (Hb ≥11 g/dL) and iron replete (ferritin >300 μg/L and TSAT >25%) at baseline, and has described a similar change in eGFR in both treatment groups over the study period [29]. This similarity was observed despite elevated iron indices in the IV iron group at month 12 (mean ferritin 363 μg/L versus 125 μg/L in the oral iron group; TSAT 30% versus 21%). However, the study analyzed only 85 patients, such that a relatively small effect on renal function may have remained undetected, and the study protocol specified only modest doses of iron supplementation in both the IV and oral iron groups (the actual amount administered was not specified). Lastly, a recent randomized trial of IV iron sucrose versus oral iron showed no change in measured GFR over 2 years’ follow-up between the two arms of the study [24]. Other randomized trials comparing IV iron versus oral iron have not reported renal function, but there was no evidence of a higher rate of renal adverse events in the IV iron groups versus patients receiving oral iron therapy [7–9, 11].

Regarding a possible effect of IV iron on proteinuria, in a blinded, randomized, placebo-controlled cross-over study of eight patients with ND-CKD, Leehey et al. assessed the effect of a single dose of ferric gluconate at a dose of 125 mg iron infused over one hour, or 250 mg iron over two hours [21]. They observed no evidence of acute renal injury, as assessed by albuminuria, proteinuria or enzymuria, although plasma levels of the oxidative stress marker malondialdehyde (MDA) increased with both doses. Another randomized trial, by Agarwal and colleagues, administered a single dose of 100 mg iron sucrose to 20 subjects with CKD stage 3 or 4, and also found an increase in MDA versus controls, accompanied by transient proteinuria and enzymuria which resolved within 24 h [22]. Similar analyses have been performed in repeated dose studies. In a multicenter, randomized trial, 62 patients with ND-CKD and iron deficiency anemia received a weekly dose of either iron sucrose or ferric gluconate (100 mg) for 5 weeks [19]. Basal levels of proteinuria were similar, but increased post-dosing, with a greater increase with iron sucrose than ferric gluconate [19]. This was consistent with results from an earlier single-dose study from the same group which showed that a single dose of iron sucrose (100 mg) provoked a significantly higher urinary protein to creatinine ratio than ferric gluconate [20]. The difference between iron sucrose and ferric gluconate is somewhat unexpected, since the latter is considered to be less stable. Other authors have reported that rapid infusion (5 min) of iron dextran or iron sucrose results in generation of reactive oxygen species [15], whereas a study of 20 iron-replete dialysis patients found that slow infusion (60 min) of these preparations did not affect biomarkers of oxidative stress or inflammation [30] (neither study measured renal function). Finally, in the prospective REVOKE trial [24], which was designed to detect renal toxicity of IV iron, proteinuria was similar in the IV iron sucrose and oral iron arms.

In the current study, FCM-treated patients received an initial single dose of up to 1000 mg iron in the high ferritin group, or 200 mg in the low ferritin group (each infused over 15 min), with mean total doses of ~2800 mg iron and ~1200 mg iron, respectively, among patients with eGFR data available at baseline and month 12. A *post hoc* analysis indicated that within this range, there was no association between quartiles of FCM dose and the change in eGFR during the 12 months after the start of FCM therapy. Moreover, neither absolute levels of ferritin (a marker for iron stores) nor TSAT (a marker for iron availability) at month 12, nor the change in ferritin or TSAT during the study, showed an association with change in eGFR. Thus, the significant increase in ferritin levels achieved in the cohort of patients randomized to high ferritin FCM was not associated with a change in renal function.

## Conclusions

The main limitation of these findings is the 1-year duration of the FIND-CKD trial which, while longer than most previous comparative studies of IV versus oral iron [7–11, 24], may not be adequate to detect a long-term effect on renal function. Within the full study cohort, we restricted the analysis to the patients who remained on the randomized study drug for the full 12-month study; any patient in whom another anemia therapy was introduced or who discontinued the study prematurely was excluded. While this reduced the population size, we believe that this was the most rigorous analytical approach. No patient discontinued the study due to decreasing renal function so bias due to selective discontinuation can be ruled out. Moreover, baseline and month 12 values for eGFR (and the extent of change from baseline to month 12) were similar in each treatment group to those seen in the full ITT population [25]. A *post hoc* repeated measured modeling calculation showed that the population analyzed here (*n* = 353) had a 15 and 18% power, respectively, to detect a difference of 1.0 ml/min/1.73 m^2^ in the change in eGFR between the high and low ferritin FCM groups, and between the high ferritin FCM and oral iron groups (40 and 49%, respectively, to detect a difference of 2 mL/min/1.73 m^2^). The study used eGFR as the indicator of renal function rather than a method to measure GFR. Furthermore, GFR estimates were based on locally determined serum creatinine values, so that variability between methods at different sites cannot be excluded. It is unlikely, however, that such variations would have obscured differences between treatment groups, since each patient served as their own control at baseline using the same assay. Another limitation is that urinary protein excretion and biomarkers of renal tubular toxicity were not recorded. Also, patients previously showing a rapidly progressive loss of renal function at screening were excluded from the study. Indeed, it is remarkable that the annual loss of eGFR was no more than 1.6 mL/min/1.73^2^ in any group. Other eligibility criteria for the study may have contributed to this stability, notably exclusion of patients with previous eGFR loss >12 mL/min/1.73 m^2^/year but also, for example, those with poorly controlled hypertension. Moreover, approximately three-quarters of patients were receiving an ACE inhibitor or an angiotensin II receptor antagonist. Lastly, in terms of the study design, the absence of a placebo arm precludes a comparison of renal function using IV iron versus no treatment and would have contributed to understanding if the observed stability of renal function was partly a trial effect.

In conclusion, results from this study indicate a lack of renal toxicity of IV iron therapy in patients with relatively stable renal function. It is important to note that these results do not necessarily apply to other IV iron preparations, due to varying molecular structures and physiochemical properties, or to patients with other characteristics (for example those receiving dialysis). Assessment of longer-term effects of IV iron, however, is required.

## Appendix 1

### The FIND-CKD Investigators

**Australia**: Simon D Roger (Gosford), Alastair Gilles (Newcastle), Randall Faull (Adelaide), Nigel D Toussaint (Parkville), Lawrence McMahon (Box Hill), Michael Suranyi (Liverpool), David Mudge (Brisbane), Brian Hutchison (Perth), Ashley Irish (Perth), Peter Kerr (Clayton), Hemant Kulkarni (Perth and Armadale), Grahame Elder (Westmead), Margaret Jardine (Concord); **Austria**: Karl Lhotta (Feldkirch), Gert Mayer (Innsbruck); **Belgium**: Raymond Vanholder (Gent), Bart Dirk Maes (Roeselare), Pieter Evenepoel (Leuven), Frédéric Debelle (Baudour), Michel Jadoul (Brussels), Max Dratwa (Brussels); **Czech Republic**: Igor Macel (Zdar nad Sazavou), Milan Dunaj (Litomysl), Milan Kvapil (Praha), Petr Bucek (Frydek-Mistek), Jitka Rehorova (Brno), Ales Hruby (Slavkov u Brna), Václava Honová (Pizen), Lada Malanova (Pizen), Martin Lucak (Prague), Dalibor Lecian (Praha), Martin Jirovec (Marianske Lazne), Jiri Vlasak (Sokolov), Ivan Rychlik (Sokolov), Stanislav Surel (Brno); **Denmark**: Anne-Lise Kamper (Kobehavn), Ove Ostergaard (Roskilde), Gudrun K Steffensen (Frederica); **France**: Leila Chenine (Montpellier), Gabrial Choukroun (Amiens), Philippe Zaoui (Grenoble); **Germany**: Christoph Wanner (Würzburg), Wolfgang Backs (Hamburg), Uwe Kraatz (Demmin), Frank Dellanna (Düsseldorf), Klaus Busch (Dortmund), Tobias Marsen (Köln), Wolfgang Seeger (Berlin), Rainer Woitas (Bonn), Nicholas Obermueller (Frankfurt/Main), Thomas Haak (Bad Mergentheim), Stephan Lueders (Cloppenburg), Frank Pistrosch (Hoyerswerda), Eckhard Mueller (Benkastel-Kues), Peter R Mertens (Magdeburg), Werner Sutermer (Würzburg), Scott-Oliver Grebe (Wuppertal), Syrus Hafezi-Rachti (Mannheim-Käfertal), Silke Roeser (Eberswalde); **Greece**: Dimitrios Tsakiris (Thessaloniki), Dimitrios Memmos (Thessanloniki), Demetrios Vlachakos (Chaidari, Athens), Vassilis Vargemezis (Dragana, Alexandroupolis), Ioannis Stefanidis (Mezourlo, Larissa), Christos Syrganis (Volos), Polichronis Alivanis (Rhodes), Ioannis Papadakis (Athens), Nickolaos Papagalanis (Athens), Aimilios Andrikos (Joannina), Dimitrios Goumenos (Rios Patras), Kostas Siamopoulos (Ioannina), Charikelia Gouva (Arta), Gabriel Papadakis (Peireus), Ioannis Boletis (Athens), Myrsini Tsimnadi-Spanoudaki (Vestos), Dimitrios Stamatiades (Serres), Kyriaki Stamatelou (Athens), Spyridon Moutafis (Athens); **Italy**: Francesco Locatelli (Lecco), Antonio Santoro (Bologna), Francesco Quarello (Torino), Giuseppe Remuzzi (Bergamo), Salvatore Coppola (Piedmonte Matese), Rosella Ferraro Mortellaro (Dan Daniele del Friuli), Andrea Icardi (Arenzano), Giacomo Colussi (Milan), Franco Della Grotta (Anzio), Luigi Lombardi (Ctanzaro), Maurizio Gallieni (Milano), Giuseppe Villa (Pavia), Giuseppe Grandaliano (Foggia); **The Netherlands**: Carlo Gaillard (Amersfoort and Amsterdam), Sebastiaan Huisman (Delft), Jos Barendregt (Apeldoorn), Peter JH Smak Gregoor (Dordrecht); **Norway**: Cecilia Oien (Trondheim); **Poland**: Boleslaw Rutkowski (Gdansk), Robert Malecki (Warszawa), Michal Nowicki (Lodz), Przemyslaw Rutkowski (Starogard Gdanski), Kryzsztof Marczewski (Zamosc), Michal Mysliwiec (Bialystok), Antoni Sydor (Tarnow), Jacek Rysz (Lodz), Andrzej Rydzewski (Warszawa), Marian Klinger (Wroclaw), Rafal Wnuk (Dabrowa Gornicza), Piotr Kozminski (Mlawa), Anna Nocon (Wroclaw), Kazimierz Ciechanowski (Szczecin); **Portugal**: Pedro Correia (Amadora), Fernando Neves (Lisboa), José Barata (Carnaxide); **Romania**: Gabriel Mircescu (Bucuresti), Mihai Voiculescu (Bucuresti), Gheorghe Gluhovschi (Timisoara), Eugen Mota (Craiova); **Spain**: Angel Luís Martín De Francisco (Santander), Alberto Torre (Madrid), Alba Herreros (Barcelona), José Luño (Madrid), E Gruss (Alcorcón), Judith Martins (Getafe [Madrid]), Marti Vallés (Girona), Julio Pascual (Barcelona); **Sweden**: Peter Bárány (Stockholm); **Switzerland**: Andreas H Bock (Aarau), Patrice M Ambuehl (Zürich); **Turkey**: Sehsuvar Erturk (Ankara), Mustafa Arici (Ankara), Saime Paydas (Adnana), Zeki Soypacaci (Izmir), Taner Camsari (Izmir), Sedat Ustundag (Edirne); **United Kingdom**: Iain C Macdougall (London), Mark E Thomas (Birmingham), Richard J D’Souza (Exeter), Jo E Taylor (Dorchester), Nicholas R Pritchard (Cambridge), Robin Jeffery (Bradford), Stephen G Riley (Cardiff), Deepak Bhatnagar (Oldham), Sunil Bhandari (Hull), David Reaich (Middlesborough), Paul E Stevens (Canterbury), Mohsen El Kossi (Doncaster), Simon Roe (Nottingham), Brian Camilleri (Ipswich), Aimun Ahmed (Preston), Arif Khwaja (Sheffield), Barbara Thompson (Stevenage), Debasish Banerjee (London), Johann Nicholas (Wolverhampton), Alistair Hutchison (Manchester), Richard Borrows (Birmingham).

## Appendix 2

**Table 3:** FIND-CKD trial: Ethics Committee approvals

Country	Site Nr	Ethic Committee
Australia	0101	Bellberry HREC 229 Greenhill Road Dulwich SA 5O65
Australia	0102	Bellberry HREC 229 Greenhill Road Dulwich SA 5O65
Australia	0103	Hunter Area Research Ethics Committee John Hunter Hospital Lookout Road New Lambton Heights NSW 23O5
Australia	0104	Research Ethics Committee Royal Adelaide Hospital North Terrace Adelaide SA 5OOO
Australia	0105	Human Research Ethics Committee Royal Melbourne Hospital Parkville, Victoria 3O5O
Australia	0106	Eastern Health HREC Level 2, 5 Arnold Street Box Hill, Victoria 3f28
Australia	0106	Eastern Health HREC Level 2, 5 Arnold Street Box Hill, Victoria 3f29
Australia	0107	Hunter Area Research Ethics Committee John Hunter Hospital Lookout Road New Lambton Heights NSW 23O5
Australia	0108	PAH Human Research Ethics Committee Tafe 3, Level 2, Bldg 35 Princess Alexandra Hospital Ispswich Road Woolloongabba, QLD 4fO2
Australia	9	Ballarat Health Services and St. John of God Health Care Ethics Committee Base Hospital Drummond Street North PO Box 577 Ballarat 3353
Australia	0110	Hunter Area Research Ethics Committee John Hunter Hospital Lookout Road New Lambton Heights NSW 2305
Australia	0111	Hunter Area Research Ethics Committee John Hunter Hospital Lookout Road New Lambton Heights NSW 2306
Australia	0111	Royal Brisbane and Women’s Hospital HREC University of Queensland, Centre for Clinical Research, Level 4, RBWH HERSTON, QUEENSLAND AUSTRALIA 4029
Australia	0112	Sir Charles Gairdner HREC Level 2, A block Hospital Avenue Nedlands, WA 6009
Australia	0113	Royal Perth Hospital HREC Colonial House Wellington Street, WA 6000
Australia	0114	Southern Health HREC 246 Clayton Road Clayton, Victoria 3168
Australia	0115	Sir Charles Gairdner HREC Level 2, A block Hospital Avenue Nedlands, WA 6009
Australia	0117	Cairns Base Hospital Ethics Committee PO Box 902 Cairns, QLD 4870
Australia	0118	Hunter Area Research Ethics Committee John Hunter Hospital Lookout Road New Lambton Heights NSW 2305
Australia	0119	Hunter Area Research Ethics Committee John Hunter Hospital Lookout Road New Lambton Heights NSW 2305
Australia	0120	Sir Charles Gairdner HREC Level 2, A block Hospital Avenue Nedlands, WA 6009
Austria	0202	Ethikkommission der Stadt Wien Town Thomas-Klestil-Platz 8/2 A-1030 Wien, Osterreich
Austria	0203	Ethikkommission der Stadt Wien Town Thomas-Klestil-Platz 8/2 A-1030 Wien, Osterreich
Austria	0204	Ethikkommission des Landes Vorarlberg Rathausstrassed 15 A-6900 Bregenz Osterreich
Austria	0205	Ethikkommission Krankenhaus der Elisabethinen Linz GmbH Fadingerstrasse 1 A-4 Linz Osterreich
Austria	0206	Ethikkommission der Medizinischen Universitat Innsbruck Innrain 43 A-6020 Innsbruck Osterreich
Austria	0207	EK des Landes Oberosterreich Landesnervenklinik Wagner-Jauregg Strasse Wagner-Jauregg Weg 15 A-4020 Linz Osterreich
Belgium	0301	Secretariaat Ethische Commissie UZ Gent Attn. Prof. Dr Matthys De Pintelaan 185 9000 Gent
Belgium	0302	H.-Hartziekenhuis Roeselare-Menen vzw Attn. Dr. Ludo Marcelis WILGENSTRAAT 2 8800 ROESELARE
Belgium	0303	Dr Van Vlem Onze-Lieve-Vrouwziekenhuis Attn. Greet de Geest Moorselbaan 164 9300 Aalst
Belgium	0304	Commissie Medische Ethiek van Universitaire Ziekenhuizen K.U.Leuven Attn. Prof. Walter Van den Bogaert Campus Gasthuisberg E330 Herestraat 49 B-3000 Leuven
Belgium	0305	Kristien Schoenmakers gang beheer en directie ZOL Campus St Jan Schiepse bos 6 3600 Genk
Belgium	0306	Hopitaux IRIS Sud-site Joseph Bracops Rue Dr Huet 79 Brussels 1070
Belgium	0307	Comité d’Ethique du Epicura Ath-Baudour Attn. Dr Frederic Debelle 136 rue Louis Caty 7331 Baudour
Belgium	0308	Commission d’Ethique Biomédicale Hospitalo- Facultaire Attn. Pr Jean-Marie Maloteaux Cliniques Universitaires Saint-Luc Avenue Hippocrate 55/14 B-1200 Bruxelles
Belgium	0309	Comite d’Ethique Hospitalo-Facultaire Universitaire de Liege Centre Hospitalier Universitaire du Sart Tilman, B35 4000 Sart Tilman par Liege 1
Belgium	0311	Centre Hospitalier Universitaire Brugman Attn. Valsamis Joseph Place A. Van Gehuchten, 4 1020 Bruxelles --2
Belgium	0312	Comite d’Ethique Clinique Universitaire de Bruxelles Hopital Erasme Route de Lennik 808 1070 Bruxelles - 7
Czech Republic	0401	Etická komise IKEM a FN Thomayerovy s poliklinikou Vídeňská 800 140 59 Praha 4
Czech Republic	0402	Etická komise pro multicentrická hodnocení Fakultní nemocnice v Motole V Úvalu 84, 150 06 Praha 5
Czech Republic	0403	Etická komise Litomyšlská nemocnice a.s. J. E. Purkyně 652 570 14 Litomyšl
Czech Republic	0404	Etická komise Nemocnice Jihlava Vrchlického 59 586 01 Jihlava
Czech Republic	0405	Etická komise pro multicentrická hodnocení Fakultní nemocnice v Motole V Úvalu 84, 150 06 Praha 5
Czech Republic	0406	Etická komise Krajská nemocnice T. Bati a.s. Zlín Havlíčkovo nábřeží 600 762 75 Zlín
Czech Republic	0407	Etická komise Fakultní nemocnice Hradec Králové Sokolská 581500 05 Hradec Králové
Czech Republic	0408	Etická Komise Nemocnice Písek, a.s. Karla Čapka 589 397 23 Písek
Czech Republic	0409	Etická komise Nemocnice Tábor, a.s. Kpt. Jaroše 2000 390 03 Tábor
Czech Republic	0410	Etická komise Nemocnice ve Frýdku-Místku, p.o. El. Krásnohorské 321 738 18 Frýdek-Místek
Czech Republic	0411	Etická komise FN Brno Bohunice Jihlavská 20 625 00 Brno
Czech Republic	0412	Etická komise B. Braun Avitum Bulovka Budínova 67 181 02 Praha 8
Czech Republic	0413	Etická komise B. Braun Avitum Bulovka Budínova 67 181 02 Praha 8
Czech Republic	0414	Etická komise Nemocnice s poliklinikou v Novém Jičíně, p.o. K Nemocnici 775/76 741 01 Nový Jičín
Czech Republic	0415	Etická komise B. Braun Avitum Bulovka Budínova 67 181 02 Praha 8
Czech Republic	0416	Etická komise Nemocnice Znojmo MUDr. Jana Janského 11 669 02 Znojmo
Czech Republic	0417	Etická komise B. Braun Avitum Bulovka Budínova 67 181 02 Praha 8
Czech Republic	0418	Etická komise společnosti Fresenius Medical Care - DS, s.r.o. Lužná 591 160 05 Praha 6
Czech Republic	0419	Etická komise pro multicentrická hodnocení Fakultní nemocnice v Motole V Úvalu 84 150 06 Praha 5
Czech Republic	0420	Etická komise společnosti Fresenius Medical Care - DS, s.r.o. Lužná 591 160 05 Praha 6
Czech Republic	0421	Etická komise společnosti Fresenius Medical Care - DS, s.r.o. Lužná 591 160 05 Praha 6
Czech Republic	0422	Etická komise společnosti Fresenius Medical Care - DS, s.r.o. Lužná 591 160 05 Praha 6
Czech Republic	0423	Etická komise společnosti Fresenius Medical Care - DS, s.r.o. Lužná 591 160 05 Praha 6
Czech Republic	0424	Etická komise pro multicentrická hodnocení Fakultní nemocnice v Motole V Úvalu 84, 150 06 Praha 5
Denmark	0501	De Videnskabsetiske Komiteer for Region Hovedstaden Regionsgarden Kongesn Vaenge CK-3400 Hillerod
Denmark	0502	De Videnskabsetiske Komiteer for Region Hovedstaden Regionsgarden Kongesn Vaenge CK-3400 Hillerod
Denmark	0503	De Videnskabsetiske Komiteer for Region Hovedstaden Regionsgården Kongens Vænge 2 DK-3400 Hillerød
Denmark	0504	De Videnskabsetiske Komiteer for Region Hovedstaden Regionsgarden Kongesn Vaenge CK-3400 Hillerod
France	0601	CPP Sud-Méditerranée IV Dr Alain DUBOIS Hopital Saint Eloi Rue Bertin Sand 34295 Montpellier Cedex 5
France	0601	CPP Sud-Méditerranée IV Dr Alain DUBOIS Hopital Saint Eloi Rue Bertin Sand 34295 Montpellier Cedex 5
France	0602	CPP Sud-Méditerranée IV Dr Alain DUBOIS Hôpital Saint Eloi Rue Bertin Sans 34295 Montpellier Cedex 5
France	0603	CPP Sud-Méditerranée IV Dr Alain DUBOIS Hôpital Saint Eloi Rue Bertin Sans 34295 Montpellier Cedex 5
France	0604	CPP Sud-Méditerranée IV Dr Alain DUBOIS Hôpital Saint Eloi Rue Bertin Sans 34295 Montpellier Cedex 5
France	0605	CPP Sud-Méditerranée IV Dr Alain DUBOIS Hôpital Saint Eloi Rue Bertin Sans 34295 Montpellier Cedex 5
France	0606	CPP Sud-Méditerranée IV Dr Alain DUBOIS Hôpital Saint Eloi Rue Bertin Sans 34295 Montpellier Cedex 5
France	0607	CPP Sud-Méditerranée IV Dr Alain DUBOIS Hôpital Saint Eloi Rue Bertin Sans 34295 Montpellier Cedex 5
France	0608	CPP Sud-Méditerranée IV Dr Alain DUBOIS Hôpital Saint Eloi Rue Bertin Sans 34295 Montpellier Cedex 5
France	0609	CPP Sud-Méditerranée IV Dr Alain DUBOIS Hôpital Saint Eloi Rue Bertin Sans 34295 Montpellier Cedex 5
France	0610	CPP Sud-Méditerranée IV Dr Alain DUBOIS Hôpital Saint Eloi Rue Bertin Sans 34295 Montpellier Cedex 5
France	0611	CPP Sud-Méditerranée IV Dr Alain DUBOIS Hôpital Saint Eloi Rue Bertin Sans 34295 Montpellier Cedex 5
France	0612	CPP Sud-Méditerranée IV Dr Alain DUBOIS Hôpital Saint Eloi Rue Bertin Sans 34295 Montpellier Cedex 5
Germany	0701	Ethik-Kommmission bei der Medizinischen Fakultät der Universitat Wurzburg Institut für Pharmakologie und Toxikologie Versbacher Str. 9 97078 Wurzburg Wuerzburg
Germany	0702	Site 0702 Ethik-Kommission der Arztekammer Hamburg Humboldtstr. 67a 22083 Hamburg
Germany	0703	Site 0703 Wthikkommission an der Medizinischen Fakultat Ernst-Moritz-Arndt-Universitat Greifswald Institut fur Pharmakologie Friedrich-Loeffler-Str. 23d 17487 Greifswald
Germany	0704	Site 0704, 0721 Ethikkommission der Arztekammer Nordrhein Tersteegenstr. 9 40474 Dusseldorf
Germany	0705	Site 0705, 0708, 0710, 0711 Ethikkommission der Arztekammer Westfalen-Lippe und der Medizinischen Fakultat der WWU-Munster Von-Esmarch-Strasse 62 48149 Munster
Germany	0706	Site 0706 Ethikkommission der Universitat Ulm Helmholtzstrasse 20 (Oberer Eselsberg) 89081 Ulm
Germany	0708	Site 0705, 0708, 0710, 0711 Ethikkommission der Arztekammer Westfalen-Lippe und der Medizinischen Fakultat der WWU-Munster Von-Esmarch-Strasse 62 48149 Munster
Germany	0709	Site 0709, 0729 Ethik-Kommission der Landesarztekammer Hessen Im Vogelsang 3 60488 Frankfurt am Main
Germany	0710	Site 0705, 0708, 0710, 0711 Ethikkommission der Arztekammer Westfalen-Lippe und der Medizinischen Fakultat der WWU-Munster Von-Esmarch-Strasse 62 48149 Munster
Germany	0711	Site 0705, 0708, 0710, 0711 Ethikkommission der Arztekammer Westfalen-Lippe und der Medizinischen Fakultat der WWU-Munster Von-Esmarch-Strasse 62 48149 Munster
Germany	0712	Site 0712, 0726 Ethik-Kommission der Bayerischen Landesarztekammer Muhlbaurstrasse 16 81677 Munchen
Germany	0713	Site 0713, 0725 Landesamt fur Gesundheit und Soziales Geschaftsstelle der Ethik-Kommission des Landes Berlin Fehrbelliner Platz 1 10707 Berlin
Germany	0714	Site 0714, 0722 Ethikkommission Landesarztekammer Rheinland-Pfalz Deutschhausplatz 3 55116 Mainz
Germany	0715	Site 0715 Ethikkommission an der Med. Fakultat der Rheinischen Friedrich-Wilhelms-Universitat Bonn Biomedizinisches Zentrum Sigmund-Freud-Str. 25 53105 Bonn
Germany	0716	Site 0716 Ethik-Kommission des Fachbereichs Medizin der Johann Wolfgang Goethe- Universitat Haus 1 Theodor-Stern-Kai 7 60590 Frankfurt
Germany	0717	Site 0717, 0730 Ethik-Kommission bei der Landesarztekammer Baden-Wurttemberg Jahnstr. 40 70597 Stuttgart
Germany	0719	Site 0719 Ethikkommission bei der Arztekammer Niedersachsen zur Beurteilung Medizinischer Forschung am Menschen Berliner Allee 20 30175 Hannover
Germany	0720	Site 0720 Ethikkommission bei der Sachsischen Landesarztekammer Schutzenhohe 16 99 Dresden
Germany	0721	Site 0704, 0721 Ethikkommission der Arztekammer Nordrhein Tersteegenstr. 9 40474 Dusseldorf
Germany	0722	Site 0714, 0722 Ethikkommission Landesarztekammer Rheinland-Pfalz Deutschhausplatz 3 55116 Mainz
Germany	0724	Site 0724 Ethik-Kommission der Otto-von-Guericke- Universitat an der Medizinischen Fakultat Leipziger Str. 44 39120 Magdeburg
Germany	0725	Site 0713, 0725 Landesamt fur Gesundheit und Soziales Geschaftsstelle der Ethik-Kommission des Landes Berlin Fehrbelliner Platz 1 10707 Berlin
Germany	0726	Site 0712, 0726 Ethik-Kommission der Bayerischen Landesarztekammer Muhlbaurstrasse 16 81677 Munchen
Germany	0727	Site 0727 Ethik-Kommission der Universitat Witten/Herdecke Alfred-Herrhausen-Str. 50 58448 Witten
Germany	0728	Site 0728 Ethikkommission der Med. Fakultat der Universitat zu Koln Gebaude 5 Kerpener Str. 62 50937 Koln
Germany	0729	Site 0709, 0729 Ethik-Kommission der Landesarztekammer Hessen Im Vogelsang 3 60488 Frankfurt am Main
Germany	0730	Site 0717, 0730 Ethik-Kommission bei der LandesarztekammerBaden-Wurttemberg Jahnstr. 40 70597 Stuttgart
Germany	0731	Site 0731 Ethik-Kommission der Landesarztekammer Brandenburg Dreifertstrasse 12 03044 Cottbus
Greece	0801	Ethical Committee General University Hospital of Thessaloniki “Papgeorgiou” Thessaloniki Ring Road, Nea Efkarpia Thessaloniki, 56429
Greece	0802	General Hospital of Thessaloniki “Ippokrateion” 49 Konstantinoupoleos st. Thessaloniki, 56442
Greece	0803	ATTIKON General University Hospital of Athens 1 Rimini Str. Chaidari, Athens, 12462
Greece	0804	General University Hospital of Alexandroupolis Dragana Alexandroupolis, 68100
Greece	0805	Ethical Commitee General University Hospital of Larissa Mezourlo Larissa, 41110
Greece	0806	Ethics Committee Achillopoulio General Prefecture Hospital of Volos 134 Polyeri street Volos, 38222
Greece	0807	Ethical Commitee General Hospital of Rhodes Aghioi Aphostoloi Rhodes, 85100
Greece	0808	Ethical Commitee IPPOKRATEION General Hospital of Athens 114 Vas. Sofias Ave Athens, 11526
Greece	0809	Ethical Commitee General Hospital of Athens, KORGALENEIO- BENAKEIO Athenasaki Str. 1 Athens, 11526
Greece	0810	Ethical Commitee General Prefecture Hospital of Ioannina, XATZIKOSTA Avv. Makrigianni 1 Ioannina, 45550
Greece	0811	Ethical Commitee General University Hospital of Patras Rio-Patras Street Rios Patras, 16500
Greece	0812	Ethical Commitee General University Hospital of Ioannina Stavros Niarchos Avenue Ioannina, 45550
Greece	0813	Ethical Commitee General Hospital of Arta A. Zara Str 4 Arta, 47100
Greece	0815	Ethical Commitee General Hospital of Peireus “Tzaneio” Zanni & Afendouli Peireus, 18536
Greece	0817	Ethical Commitee General Prefecture Hospital of Argos 191 Korinthou Str. Argos, 21200
Greece	0818	Ethical Commitee LAIKO General Hospital of Athens 17 Aghiou Thomas Str. Athens, 11527
Greece	0819	Ethical Commitee General Hospital of Mytilene “Vostanio” 48 E. Vostani Str. Vestos, 81100
Greece	0820	Ethical Commitee General Hospital of Serres 2nd k of Serres-Drama National Road Serres, 62100
Greece	0821	Ethical Commitee KYANOUS STAVROS General Hospital of Athens 102, Vas Sofias Ave Athens, 11528
Greece	0822	Ethical Commitee IASO General Hospital of Athens Cholargos Athens, 11526
Greece	0823	Ethical Commitee General Hospital of Athens “Henry Dunant” 107 Messogheion Ave Athens, 11526
Italy	1001	Comitato Etico Dell’Azienda Ospedaliera di Lecco Via Dell’Eremo 9/11 Lecco, 23900
Italy	1002	Comitato Etico Locale per la Sperimentazione Clinical Della AUSL 12 di Viareggio Via Aurelia 335 55045 Lido di Cà Maiore (LC)
Italy	1003	Comitato Etico Dell’Azeinda Ospedaliera Universitaria Della Seconda Univestità degli Studi di Napoli Via Costatinopoli, 104 80138 Napoli
Italy	1004	Comitato Etico Indipendente dell’Azienda ospedaliero-Univesitaria Policlinico S. Orsola Via Albertoni 15 40138 Bologna
Italy	1005	Comitato Etico Della ASL TO/2 di Torino Corso Svizzera 185 bis 10149 Torino
Italy	1007	Comitato di Bioetica della Azienda Ospedali Riuniti di Bergam Largo Barozzi 1 24128 Bergamo
Italy	1008	Comitato Etico ASL di Caserta Via Unità Italiana 28 81100 Caserta
Italy	1009	Comitato Etico Dell’Azienda Ospedaliera Universitaria ‘S. Martin’ di Genova Largo Rosanna Benzi 10 16132 Genova
Italy	1	Comitato Etico Della Provincia di Modena Via Largo del Pozzo 71 41124 Modena
Italy	1011	Comitato Etico ASL CE/1 Di Caserta Via Unità Italiana 28 81100 Caserta
Italy	1012	Comitato Etico Regionale Unico (CERU) AOU Santa Maria della Misericordia Piazzale Santa maria della Misericordia 15 33100 Udine
Italy	1013	Comitato Etico Scientifico Dell’Azienda Ospedaliera Ospedale S. Carlo Borromeo di Milano Via Pio II° n° 3 20153 Milano
Italy	1014	Comitato Bioetico Dell’Azienda Cannizzaro di Catania Via Messina 829 95126 Catania
Italy	1016	Comitato Ethico-Scientifico Dell’Azienda Ospedaliera Ospedale Niguara Ca’ Granda Di Milano Piazza Ospedale Maggiore n. 3 20162 Milano
Italy	1017	Comitato Etico Della AUSL RM/H Di Albano Laziale Borgo Garibaldi n. 12 00041 Albano Laziale (RM)
Italy	1018	Comitato Etico Dell’Azienda Ospedaliera Pugliese-Ciaccio Di Catanzaro Via Vinicio Cortese, 10 88100 Catanzaro
Italy	1020	Comitato Etico Dell’Azienda Ospedaliera Universitaria S. Giovanni Battista di Torino C so Bramante 88/90 10126 Torino
Italy	1021	Comitato Etico Scientifico Dell’Azienda Ospedaliera Ospedale S. Carlo Borrome di Milano Via Pio II°, n°3 20153 Milano
Italy	1022	Comitato Etico Central Dell’IRCCS Fondazione Salvatore Maugeri Di Pavia Via Salvatore Maugeri 4 27100 Pavia
Italy	1023	Comitato Etico Sperimentazione clinical Medicinali Della AUSL 8 Di Arezzo Via Curtatone 54 52100 Arezzo
Italy	1024	Comitato Etico Azienda Ospedaliera Universitaria Ospedali Riuniti di figgia Viale Luigi Pinto 71100 Foggia
Italy	1026	Comitato di Etica Della ASL di Salerno Via Federico Ricco, 50 84014 Noceria Inferiore (SA)
Italy	1027	Comitato Etico Per le Sperimentazioni Cliniche die Medicinali Della Provincia di Venezia Via Don Federico Tosatto 147 30174 Venezia
Italy	1028	Comitato Etico Della AUSL RM/G di Tivoli Via Tiburtina 22/a 00019 Tivoli (RM)
Italy	1029	Comitato Etico Delle Aziende Sanitarie Dell’Umbria di Perugia Via della Rivoluzione 16 Ellera di Corciano (PG) 06070 Perugia
Netherlan ds	1101	Meander Medical Center, Lichtenberg location Toetsingscommissie Wetenschappelijk Onderzoek Secretariat, P&O Room N042 Utrechtseweg 160 3818 ES Amersfoort The Netherlands
Netherlan ds	1102	Medical Ethics Review Committee Zuidwest Holland Fonteynenburghlaan 7 2275 CX VOORBURG The Netherlands
Netherlan ds	1102	Medical Ethics Review Committee Gelre Hospital Albert Schweitzerlaan 31 7334 DZ Apeldoorn The Netherlands
Netherlan ds	1103	Medical Ethics Review Committee Gelre Hospital Albert Schweitzerlaan 31 7334 DZ Apeldoorn The Netherlands
Netherlan ds	1104	Medical Ethics Review Committee Albert Schweitzer Hospital loc. DW, Postvak 7, kmr. Z 150 T.a.v. Ms. A. de Graag – de Vries Albert Schweitzerplaats 25 3318 AT Dordrecht The Netherlands
Netherlan ds	1105	METc VU Medical Center Medical Faculty, Room H-565 Van der Boerchorststraat 7 1081 BT Amsterdam The Netherlands
Netherlan ds	1106	Medical Ethics Review Committee Noord- Holland Foreest Medical School Nassauplein 10 1815 GM Alkmaar The Netherlands
Norway	1201	Regional Committees for Medical and Health Research Ethics (REK) REK-Midt Bygg for samfunnsmedisin (5 etg) Håkon Jarlsgt. 11, St. Olavs Hospital Trondheim
Poland	1301	Niezależna Komisja Bioetyczna do Spraw Badań Naukowych przy Gdańskim Uniwersytecie Medycznym ul. M. Skłodowskiej-Curie 3a, 80-201 Gdańsk, Polska
Poland	1302	Niezależna Komisja Bioetyczna do Spraw Badań Naukowych przy Gdańskim Uniwersytecie Medycznym ul. M. Skłodowskiej-Curie 3a, 80-201 Gdańsk, Polska
Poland	1303	Niezależna Komisja Bioetyczna do Spraw Badań Naukowych przy Gdańskim Uniwersytecie Medycznym ul. M. Skłodowskiej-Curie 3a, 80-201 Gdańsk, Polska
Poland	1306	Niezależna Komisja Bioetyczna do Spraw Badań Naukowych przy Gdańskim Uniwersytecie Medycznym ul. M. Skłodowskiej-Curie 3a, 80-201 Gdańsk, Polska
Poland	1309	Niezależna Komisja Bioetyczna do Spraw Badań Naukowych przy Gdańskim Uniwersytecie Medycznym ul. M. Skłodowskiej-Curie 3a, 80-201 Gdańsk, Polska
Poland	1311	Niezależna Komisja Bioetyczna do Spraw Badań Naukowych przy Gdańskim Uniwersytecie Medycznym ul. M. Skłodowskiej-Curie 3a, 80-201 Gdańsk, Polska
Poland	1313	Niezależna Komisja Bioetyczna do Spraw Badań Naukowych przy Gdańskim Uniwersytecie Medycznym ul. M. Skłodowskiej-Curie 3a, 80-201 Gdańsk, Polska
Poland	1314	Niezależna Komisja Bioetyczna do Spraw Badań Naukowych przy Gdańskim Uniwersytecie Medycznym ul. M. Skłodowskiej-Curie 3a, 80-201 Gdańsk, Polska
Poland	1315	Niezależna Komisja Bioetyczna do Spraw Badań Naukowych przy Gdańskim Uniwersytecie Medycznym ul. M. Skłodowskiej-Curie 3a, 80-201 Gdańsk, Polska
Poland	1316	Niezależna Komisja Bioetyczna do Spraw Badań Naukowych przy Gdańskim Uniwersytecie Medycznym ul. M. Skłodowskiej-Curie 3a, 80-201 Gdańsk, Polska
Poland	1318	Niezależna Komisja Bioetyczna do Spraw Badań Naukowych przy Gdańskim Uniwersytecie Medycznym ul. M. Skłodowskiej-Curie 3a, 80-201 Gdańsk, Polska
Poland	1320	Niezależna Komisja Bioetyczna do Spraw Badań Naukowych przy Gdańskim Uniwersytecie Medycznym ul. M. Skłodowskiej-Curie 3a, 80-201 Gdańsk, Polska
Poland	1321	Niezależna Komisja Bioetyczna do Spraw Badań Naukowych przy Gdańskim Uniwersytecie Medycznym ul. M. Skłodowskiej-Curie 3a, 80-201 Gdańsk, Polska
Poland	1322	Niezależna Komisja Bioetyczna do Spraw Badań Naukowych przy Gdańskim Uniwersytecie Medycznym ul. M. Skłodowskiej-Curie 3a, 80-201 Gdańsk, Polska
Poland	1323	Niezależna Komisja Bioetyczna do Spraw Badań Naukowych przy Gdańskim Uniwersytecie Medycznym ul. M. Skłodowskiej-Curie 3a, 80-201 Gdańsk, Polska
Poland	1324	Niezależna Komisja Bioetyczna do Spraw Badań Naukowych przy Gdańskim Uniwersytecie Medycznym ul. M. Skłodowskiej-Curie 3a, 80-201 Gdańsk, Polska
Poland	1326	Niezależna Komisja Bioetyczna do Spraw Badań Naukowych przy Gdańskim Uniwersytecie Medycznym ul. M. Skłodowskiej-Curie 3a, 80-201 Gdańsk, Polska
Poland	1327	Niezależna Komisja Bioetyczna do Spraw Badań Naukowych przy Gdańskim Uniwersytecie Medycznym ul. M. Skłodowskiej-Curie 3a, 80-201 Gdańsk, Polska
Poland	1328	Niezależna Komisja Bioetyczna do Spraw Badań Naukowych przy Gdańskim Uniwersytecie Medycznym ul. M. Skłodowskiej-Curie 3a, 80-201 Gdańsk, Polska
Poland	1329	Niezależna Komisja Bioetyczna do Spraw Badań Naukowych przy Gdańskim Uniwersytecie Medycznym ul. M. Skłodowskiej-Curie 3a, 80-201 Gdańsk, Polska
Poland	1330	Niezależna Komisja Bioetyczna do Spraw Badań Naukowych przy Gdańskim Uniwersytecie Medycznym ul. M. Skłodowskiej-Curie 3a, 80-201 Gdańsk, Polska
Poland	1331	Niezależna Komisja Bioetyczna do Spraw Badań Naukowych przy Gdańskim Uniwersytecie Medycznym ul. M. Skłodowskiej-Curie 3a, 80-201 Gdańsk, Polska
Portugal	1402	CEIC- National Ethics Committee for Clinical Investigation Parque da Saúde de Lisboa- Avenida do Brasil, 53 1749-004 Lisboa- Portugal
Portugal	1403	CEIC- National Ethics Committee for Clinical Investigation Parque da Saúde de Lisboa- Avenida do Brasil, 53 1749-004 Lisboa- Portugal
Portugal	1404	CEIC- National Ethics Committee for Clinical Investigation Parque da Saúde de Lisboa- Avenida do Brasil, 53 1749-004 Lisboa- Portugal
Portugal	1405	CEIC- National Ethics Committee for Clinical Investigation Parque da Saúde de Lisboa- Avenida do Brasil, 53 1749-004 Lisboa- Portugal
Portugal	1406	CEIC- National Ethics Committee for Clinical Investigation Parque da Saúde de Lisboa- Avenida do Brasil, 53 1749-004 Lisboa- Portugal
Portugal	1407	CEIC- National Ethics Committee for Clinical Investigation Parque da Saúde de Lisboa- Avenida do Brasil, 53 1749-004 Lisboa- Portugal
Spain	1501	Hospital Universitario Dr Peset de Valencia CEIC, a/a Raquel E. Blesa, C/Juan de Garray 21, 1er Piso Consultas externas, 46017 Valencia
Spain	1503	Hospital Universitario Dr Peset de Valencia CEIC, a/a Raquel E. Blesa, C/Juan de Garray 21, 1er Piso Consultas externas, 46017 Valencia
Spain	1504	CEIC Hospital Universitario La Paz(LEC) Paseo de la Castellana, 261, Planta 8a Hospital General, 28046 Madrid
Spain	1505	CEIC Fundació Puigvert IUNA (LEC) Agencia de Gestio del Coneixement Cartagena, 340-350 08025 Barcelona
Spain	1506	CEIC Hospital Universitario General Gregorio Marañón (CEC) CEIC Area 1, C/ dr Esquerdo, 46, 28007 Madrid
Spain	1507	Agencia de Ensayos Clinicos - servicio de Farmacia Hospital Clinic de Barcelona, c/ Villarroel, 170 - Sotano, Escalera 6b, 08036 Barcelona
Spain	1509	CEIC Hospital Universitario de Bellvitge Edificio Consultas Externas. Planta -1, C/ Feixa Llarga, s/n, 08907 L’Hospitalet de Llobregat, Barcelona
Spain	1510	CEIC Hospital Universitari Vall d'Hebron Edifici Institut de Recerca, 2a planta Passeig Vall d’Hebron 119-129, Barcelona 08035
Spain	1512	CEIC Hospital Universitario Fundación de Alcorcón (LEC) C/ Budapest N1, 28922 Alcorcon, Madrid
Spain	1513	CEIC Clinica de Asturias Hospital Central de Asturias, Celestino Villamil, s/n, 33006 Oviedo
Spain	1514	Hopsital Universitario “Reina Sofia” Comite Etico de Ensayos Clinicos, Edificio de Consultas Externas, planta -1, Avda. Menendez Pidal, s/n, 14004 Cordoba
Spain	1515	Hospital Torrecardenas CEIC Paraje Torrecardenas, s/n, 04009 Almeria
Spain	1516	Fundacion Jimenez Diaz CEIC, Avda. Reyes Catolicos, 2, Entrplanta, 28040 Madrid
Spain	1517	Hospital Universitario Principe de Asturias CEIC, Ctra. Alcala-Meco s/n, 28805 Alcala de Henares, Madrid
Spain	1518	CEIC de Aragon, Avda. Gomez Laguna, 25 planta 11, 50009 Zaragoza
Spain	1519	Hospital Universitario de Puerto Real, Ctra. NaI IV, km. 665, 11510 Puerto Real, Cadiz
Spain	1520	CEIC Hospital Universitario de Getafe (LEC) Ctra. De Toledo, km. 12500, 28905 Getafe, Madrid
Spain	1521	CEIC Hospital Universitario La Princesa, Findacion para la Investigacion Biomedica, C/ Diego de leon, 62, 28006 Madrid
Spain	1522	CEIC Hospital Universitario de Girona Josep Trueta (LEC) avda. De Franca s/n, 17007 Girona
Spain	1523	CEIC Parc Salut del Mar (LEC) IMIM-Hospital del Mar, Parc de Recerca Biomedica de Barcelona, Doctor Aiguader, 88, 08003 Barcelona
Sweden	1601	Regionala etikprövningsnämnden Stockholm FE 289 Karolinska Institutet Stockholm, 17179
Sweden	1602	Regionala etikprövningsnämnden Stockholm FE 289 Karolinska Institutet Stockholm, 17179
Sweden	1603	Regionala etikprövningsnämnden Stockholm FE 289 Karolinska Institutet Stockholm, 17179
Switzerland	1701	Kantonal Ethikkommission Aargau Departement Gesundheit und Soziales PD Dr. med. Otto Hilfiker Bachstrasse 15 5001 Aarau
Switzerlan d	1702	Kantonal Ethik-Kommission (KEK) Prof. Dr. med. Robert Maurer Universitätsspital Zürich Sonneggstr. 12 8091 Zürich
Switzerlan d	1703	Kantonal Ethik-Kommission (KEK) Prof. Dr. med. Robert Maurer Universitätsspital Zürich Sonneggstr. 12 8091 Zürich
Switzerlan d	1704	Kantonal Ethikkommission Bern (KEK) Prof. Dr. pharm. Nilaus Tüller Postfach 56 3 Bern
Turkey	1801	Ankara University Medical Faculty Deanship Clinical Researches Ethics Committee nkara Universitesi Tip Fakultesi Morfoloji Binası 06100 Sihhiye Ankara Turkey
Turkey	1802	Ege University Medical Faculty Clinical Researches Ethics Committee Ege Universitesi Tip Fakultesi; 35100 Bornova Izmir
Turkey	1803	Ege University Medical Faculty Clinical Researches Ethics Committee Ege Universitesi Tip Fakultesi; 35100 Bornova Izmir
Turkey	1804	Ege University Medical Faculty Clinical Researches Ethics Committee Ege Universitesi Tip Fakultesi; 35100 Bornova Izmir
Turkey	1804	Ege University Medical Faculty Clinical Researches Ethics Committee Ege Universitesi Tip Fakultesi; 35100 Bornova Izmir
Turkey	1807	Ege University Medical Faculty Clinical Researches Ethics Committee Ege Universitesi Tip Fakultesi; 35100 Bornova Izmir
Turkey	1810	Ege University Medical Faculty Clinical Researches Ethics Committee Ege Universitesi Tip Fakultesi; 35100 Bornova Izmir
Turkey	1811	Ege University Medical Faculty Clinical Researches Ethics Committee Ege Universitesi Tip Fakultesi; 35100 Bornova Izmir
United Kingdom	1901	Health Research Authority NRES Committee Riverside REC Bristol REC Centre Level 3, Block B Whitefriars Lewins Mead Bristol, BS 1 2NT
United Kingdom	1902	Health Research Authority NRES Committee Riverside REC Bristol REC Centre Level 3, Block B Whitefriars Lewins Mead Bristol, BS 1 2NT
United Kingdom	1903	Health Research Authority NRES Committee Riverside REC Bristol REC Centre Level 3, Block B Whitefriars Lewins Mead Bristol, BS 1 2NT
United Kingdom	1904	Health Research Authority NRES Committee Riverside REC Bristol REC Centre Level 3, Block B Whitefriars Lewins Mead Bristol, BS 1 2NT
United Kingdom	1905	Health Research Authority NRES Committee Riverside REC Bristol REC Centre Level 3, Block B Whitefriars Lewins Mead Bristol, BS 1 2NT
United Kingdom	1906	Health Research Authority NRES Committee Riverside REC Bristol REC Centre Whitefriars, Lewins Mead
United Kingdom	1907	Health Research Authority NRES Committee Riverside REC Bristol REC Centre Whitefriars, Lewins Mead
United Kingdom	1908	Health Research Authority NRES Committee Riverside REC Bristol REC Centre Whitefriars, Lewins Mead
United Kingdom	1909	Health Research Authority NRES Committee Riverside REC Bristol REC Centre Whitefriars, Lewins Mead
United Kingdom	1910	Health Research Authority NRES Committee Riverside REC Bristol REC Centre Whitefriars, Lewins Mead
United Kingdom	1911	Health Research Authority NRES Committee Riverside REC Bristol REC Centre Whitefriars, Lewins Mead
United Kingdom	1912	Health Research Authority NRES Committee Riverside REC Bristol REC Centre Whitefriars, Lewins Mead
United Kingdom	1913	Health Research Authority NRES Committee Riverside REC Bristol REC Centre Whitefriars, Lewins Mead
United Kingdom	1914	Health Research Authority NRES Committee Riverside REC Bristol REC Centre Whitefriars, Lewins Mead
United Kingdom	1915	Health Research Authority NRES Committee Riverside REC Bristol REC Centre Whitefriars, Lewins Mead
United Kingdom	1916	Health Research Authority NRES Committee Riverside REC Bristol REC Centre Whitefriars, Lewins Mead
United Kingdom	1917	Health Research Authority NRES Committee Riverside REC Bristol REC Centre Level 3, Block B Whitefriars Lewins Mead Bristol, BS 1 2NT
United Kingdom	1918	Health Research Authority NRES Committee Riverside REC Bristol REC Centre Level 3, Block B Whitefriars Lewins Mead Bristol, BS 1 2NT
United Kingdom	1919	Health Research Authority NRES Committee Riverside REC Bristol REC Centre Level 3, Block B Whitefriars Lewins Mead Bristol, BS 1 2NT
United Kingdom	1920	Health Research Authority NRES Committee Riverside REC Bristol REC Centre Level 3, Block B Whitefriars Lewins Mead Bristol, BS 1 2NT
United Kingdom	1921	Health Research Authority NRES Committee Riverside REC Bristol REC Centre Level 3, Block B Whitefriars Lewins Mead Bristol, BS 1 2NT
United Kingdom	1922	Health Research Authority NRES Committee Riverside REC Bristol REC Centre Level 3, Block B Whitefriars Lewins Mead Bristol, BS 1 2NT
United Kingdom	1923	Health Research Authority NRES Committee Riverside REC Bristol REC Centre Level 3, Block B Whitefriars Lewins Mead Bristol, BS 1 2NT
United Kingdom	1924	Health Research Authority NRES Committee Riverside REC Bristol REC Centre Level 3, Block B Whitefriars Lewins Mead Bristol, BS 1 2NT
United Kingdom	1925	Health Research Authority NRES Committee Riverside REC Bristol REC Centre Level 3, Block B Whitefriars Lewins Mead Bristol, BS 1 2NT
United Kingdom	1926	Health Research Authority NRES Committee Riverside REC Bristol REC Centre Level 3, Block B Whitefriars Lewins Mead Bristol, BS 1 2NT
United Kingdom	1927	Health Research Authority NRES Committee Riverside REC Bristol REC Centre Level 3, Block B Whitefriars Lewins Mead Bristol, BS 1 2NT
United Kingdom	1928	Health Research Authority NRES Committee Riverside REC Bristol REC Centre Level 3, Block B Whitefriars Lewins Mead Bristol, BS 1 2NT
United Kingdom	1929	Health Research Authority NRES Committee Riverside REC Bristol REC Centre Level 3, Block B Whitefriars Lewins Mead Bristol, BS 1 2NT
United Kingdom	19230	Health Research Authority NRES Committee Riverside REC Bristol REC Centre Level 3, Block B Whitefriars Lewins Mead Bristol, BS 1 2NT
United Kingdom	1931	Health Research Authority NRES Committee Riverside REC Bristol REC Centre Level 3, Block B Whitefriars Lewins Mead Bristol, BS 1 2NT
United Kingdom	1932	Health Research Authority NRES Committee Riverside REC Bristol REC Centre Level 3, Block B Whitefriars Lewins Mead Bristol, BS 1 2NT
United Kingdom	1933	Health Research Authority NRES Committee Riverside REC Bristol REC Centre Level 3, Block B Whitefriars Lewins Mead Bristol, BS 1 2NT
Romania	2001	National Ethics Committee for Clinical Study of Medicine (Comisia Nationala de Etica pentru Studiul Clinic al Medicamentului) Av. Sanatescu St., No. 48, 1st district, 011478, Bucharest, Romania
Romania	2002	National Ethics Committee for Clinical Study of Medicine (Comisia Nationala de Etica pentru Studiul Clinic al Medicamentului) Av. Sanatescu St., No. 48, 1st district, 011478, Bucharest, Romania
Romania	2003	National Ethics Committee for Clinical Study of Medicine (Comisia Nationala de Etica pentru Studiul Clinic al Medicamentului) Av. Sanatescu St., No. 48, 1st district, 011478, Bucharest, Romania
Romania	2004	National Ethics Committee for Clinical Study of Medicine (Comisia Nationala de Etica pentru Studiul Clinic al Medicamentului) Av. Sanatescu St., No. 48, 1st district, 011478, Bucharest, Romania
Romania	2005	National Ethics Committee for Clinical Study of Medicine (Comisia Nationala de Etica pentru Studiul Clinic al Medicamentului) Av. Sanatescu St., No. 48, 1st district, 011478, Bucharest, Romania
Romania	2006	National Ethics Committee for Clinical Study of Medicine (Comisia Nationala de Etica pentru Studiul Clinic al Medicamentului) Av. Sanatescu St., No. 48, 1st district, 011478, Bucharest, Romania
Romania	2007	National Ethics Committee for Clinical Study of Medicine (Comisia Nationala de Etica pentru Studiul Clinic al Medicamentului) Av. Sanatescu St., No. 48, 1st district, 011478, Bucharest, Romania
US	2110	Salem VA Medical Center IRB Kim Ragsdale, PhD 1970 Roanoke Blvd Salem. VA 24153
US	2113	Integreview IRB Valerie Nelson 3001, South Lamar Blvd Suite 210 Austin, TX 78704
US	2105	Integreview IRB Valerie Nelson 3001, South Lamar Blvd Suite 210 Austin, TX 78704
US	2114	Temple VA Medical Cener IRB John W Klocek, PhD 1901 Veterans Memorial Drive Temple, TX 76504

## Additional files

Additional file 1: Table S1. Baseline characteristics (ITT population). (DOCX 12 kb)
Additional file 2: Table S2. Mean (SD) change in estimated GFR (eGFR) from baseline to month 12 for subpopulations of patients with eGFR values both time points. (DOCX 12 kb)
Additional file 3: Figure S1. Scatter plot of change in eGFR from baseline to month 12 according to total IV iron dose in patients randomized to either high ferritin or low ferritin FCM for patients with eGFR values at baseline and month 12. The solid line indicates the linear regression for all points. The dotted line indicates 0 i.e. no change. eGFR, estimated glomerular filtration rate; FCM, ferric carboxymaltose. (EPS 1219 kb)
Additional file 4: Figure S2. (a) Ferritin and (b) transferrin saturation (TSAT) for patients with eGFR values at baseline and month 12 who did or did not receive alternative anemia therapy or permanently discontinued study therapy before month 12. BL, baseline; FCM, ferric carboxymaltose. (ZIP 456 kb)
Additional file 5: Table S3. Selected renal adverse events and serious adverse events (safety population). (DOCX 11 kb)

## Abbreviations

ACE: Angiotensin converting enzyme
ANCOVA: Analysis of covariance
ARB: Angiotensin II receptor blocker
BMI: Body mass index
CKD: Chronic kidney disease
CKD-EPI: Chronic Kidney Disease Epidemiology Collaboration
eGFR: estimated GFR
ESA: Erythropoiesis-stimulating agent
FCM: Ferric carboxymaltose
ITT: Intention-to-treat
IV: Intravenous
MDA: Malondialdehyde
MDRD: Modification of Diet in Renal Disease
ND-CKD: Non-dialysis dependent CKD
NTBI: Non-transferrin bound iron
TSAT: Transferrin saturation
