# Supplementary material for: Renal function in patients with non-dialysis chronic kidney disease receiving intravenous ferric carboxymaltose: an analysis of the randomized FIND-CKD trial
Source: BMC Nephrol. 2017 Jan 17;18:24. doi: 10.1186/s12882-017-0444-6 (PMC5240256; doi:10.1186/s12882-017-0444-6)
Supplement: Additional file 1: Table S1. — Baseline characteristics (ITT population). (DOCX 12 kb) [file 12882_2017_444_MOESM1_ESM.docx]

**Supplementary Table 1.** Baseline characteristics (ITT population)

|  | **High ferritin FCM (n=153)** | **Low ferritin FCM (n=152)** | **Oral iron (n=308)** |
| --- | --- | --- | --- |
| Age, years | 69.5 (12.6) | 68.2 (13.3) | 69.3 (13.4) |
| Female gender, n (%) | 91 (59.5) | 98 (64.5) | 192 (62.3) |
| White race, n (%) | 149 (97.4) | 144 (94.7) | 291 (94.5) |
| Body mass index, kg/m^2^ | 29.7 (6.6) | 29.9 (6.0) | 29.1 (5.9) |
| History of diabetes, n (%) | 88 (57.5) | 97 (63.8) | 195 (63.3) |
| Endogenous erythropoietin, mIU/mL | 28.2 (30.0) | 27.1 (25.0) | 31.4 (91.5) |
| Hb, g/dL | 10.3 (0.7) | 10.5 (0.8) | 10.4 (0.7) |
| Ferritin, μg/L | 57.7 (48.1) | 56.4 (49.2) | 57.3 (42.4) |
| TSAT, % | 16.2 (16.7) | 16.1 (8.3) | 15.5 (7.6) |
| C-reactive protein, mg/L | 6.7 (11.3) | 6.2 (9.1) | 5.2 (6.1) |
| eGFR, mL/min/1.73m^2^ | 32.8 (11.7) | 31.5 (10.7) | 32.3 (11.6) |
| ACE inhibitor therapy prior to study entry, n (%)^a^ | 61 (39.6) | 65 (43.3) | 125 (40.1) |
| Angiotensin II antagonist therapy prior to study entry, n (%)^b^ | 60 (39.0) | 65 (43.3) | 135 (43.3) |

Continuous variables are shown as mean (SD).
ACE, angiotensin converting enzyme inhibitor, eGFR, estimated glomerular filtration rate; FCM, ferric carboxymaltose; Hb, hemoglobin; TSAT, transferrin saturation

^a^ Includes patients receiving ACE inhibitor combinations
^b^ Includes patients receiving angiotensin II antagonist combinations
