# Supplementary material for: Renal function in patients with non-dialysis chronic kidney disease receiving intravenous ferric carboxymaltose: an analysis of the randomized FIND-CKD trial
Source: BMC Nephrol. 2017 Jan 17;18:24. doi: 10.1186/s12882-017-0444-6 (PMC5240256; doi:10.1186/s12882-017-0444-6)
Supplement: Additional file 2: Table S2. — Mean (SD) change in estimated GFR (eGFR) from baseline to month 12 for subpopulations of patients with eGFR values both time points. (DOCX 12 kb) [file 12882_2017_444_MOESM2_ESM.docx]

**Additional file 2: Table S2.** Mean (SD) change in estimated GFR (eGFR) from baseline to month 12 for subpopulations of patients with eGFR values both time points

|  | **High ferritin FCM (n=97)** | | **Low ferritin FCM (n=89)** | | **Oral iron  (n=167)** | |
| --- | --- | --- | --- | --- | --- | --- |
|  | **n** | **Mean (SD))** | **n** | **Mean (SD)** | **n** | **Mean (SD)** |
| Age, years^a^  ≤72  >72 | 49 48 | -0.8 (8.1) 2.2 (9.3) | 47 42 | -1.5 (7.2) 0.1 (8.6) | 81 86 | -0.5 (8.2) -1.0 (9.2) |
| Gender  Female  Male | 61 36 | 0.7 (6.9) 0.7 (11.4) | 56 33 | -0.4 (8.9) -1.3 (6.0) | 106 61 | -0.2 (8.9) -1.7 (8.2) |
| Body mass index, kg/m^2 a^   ≤29.3  >29.3 | 47 49 | 0.5 (11.2) 1.1 (5.7) | 39 50 | 1.5 (9.2) -2.5 (6.3) | 90 77 | 0.2 (8.9) -2.0 (8.4) |
| Diabetes  Yes  No | 61 36 | 0.2 (7.8) 1.5 (10.4) | 59 30 | -1.7 (7.7) 1.3 (8.1) | 106 61 | -1.8 (8.2) 1.0 (9.2) |
| Systolic blood pressure, mmHg^a^  ≤135  >135 | 40 57 | 4.0 (9.2) -1.6 (7.7) | 43 46 | 1.2 (8.5) -2.5 (6.9) | 95 72 | -0.4 (9.2) -1.3 (8.0) |
| Diastolic blood pressure, mmHg^a^  ≤135  >135 | 51 46 | 1.3 (8.6) 0.1 (9.0) | 45 44 | 0.2 (9.1) -1.6 (6.5) | 82 85 | -0.8 (9.6) -0.7 (7.8) |
| Mean arterial pressure, mmHg^a^  ≤93.3  >93.3 | 43 54 | 1.4 (8.6) 0.1 (9.0) | 40 49 | 1.8 (8.7) -2.8 (6.6) | 82 85 | -0.5 (9.5) -1.1 (7.8) |
| ACE inhibitor therapy prior to study entry  Yes  No | 32 65 | -0.3 (7.0 1.2 (9.6) | 37 52 | -1.9 (6.1) 0.1 (8.9) | 69 98 | -1.8 (9.2) -0.1 (8.3) |
| Angiotensin II antagonist therapy prior to study entry  Yes  No | 41 56 | 0.4 (7.7) 0.9 (9.6) | 33 56 | -0.5 (9.5) -0.9 (6.9) | 77 90 | -1.1 (8.1) -0.5 (9.2) |

ACE, angiotensin converting enzyme; FCM, ferric carboxymaltose; LS, least squares; SE, standard error ^a^ Cut-off point at median value
