# Supplementary material for: Renal function in patients with non-dialysis chronic kidney disease receiving intravenous ferric carboxymaltose: an analysis of the randomized FIND-CKD trial
Source: BMC Nephrol. 2017 Jan 17;18:24. doi: 10.1186/s12882-017-0444-6 (PMC5240256; doi:10.1186/s12882-017-0444-6)
Supplement: Additional file 5: Table S3. — Selected renal adverse events and serious adverse events (safety population). (DOCX 11 kb) [file 12882_2017_444_MOESM5_ESM.docx]

**Additional file 5: Table S3.** Selected renal adverse events and serious adverse events (safety population)

| **Adverse events** | **High ferritin FCM (n=154)** | **Low ferritin FCM (n=150)** | **FCM total (n=304)** | **Oral iron  (n=312)** |
| --- | --- | --- | --- | --- |
| Chronic renal failure | 3 (1.9) | 1 (0.7) | 4 (1.3) | 8 (2.6) |
| Renal failure | 1 (0.6) | 3 (2.0) | 4 (1.3) | 7 (2.2) |
| Renal impairment | 3 (1.9) | 2 91.3) | 5 (1.6) | 6 (1.9) |
| Acute renal failure | 2 (1.3) | 0 | 2 (0.7) | 3 (1.0) |
| Nephrotic syndrome | 1 (0.6) | 1 (0.7) | 2 (0.7) | 0 |
| Nephropathy | 0 | 1 (0.7) | 1 (0.3) | 0 |
| Proteinuria | 1 (0.6) | 0 | 1 (0.3) | 0 |
| Increased blood creatinine | 3 (1.9) | 3 (2.0) | 6 (2.0) | 5 (1.6) |
| **Serious adverse events** |  |  |  |  |
| Chronic renal failure | 1 (0.6) | 1 (0.7) | 2 (0.7) | 6 (1.9) |
| Renal failure | 1 (0.6) | 0 | 1 (0.3) | 2 (0.6) |
| Renal impairment | 0 | 0 | 0 | 2 (0.6) |
| Acute renal failure | 1 (0.6) | 0 | 1 (0.3) | 1 (0.3) |
| Nephrotic syndrome | 1 (0.6) | 1 (0.7) | 2 (0.7) | 0 |

Adverse events and serious adverse events are reported up to the point at which another anemia therapy was initiated and/or the randomized study medication was discontinued, based on clinician’s judgment

FCM, ferric carboxymaltose
